# Laser Printing of Multilayered Alternately Conducting and Insulating Microstructures

**DOI:** 10.1021/acsami.1c06204

**Published:** 2021-07-23

**Authors:** Eitan Edri, Nina Armon, Ehud Greenberg, Shlomit Moshe-Tsurel, Danielle Lubotzky, Tommaso Salzillo, Ilana Perelshtein, Maria Tkachev, Olga Girshevitz, Hagay Shpaisman

**Affiliations:** † Department of Chemistry, Bar-Ilan University, Ramat Gan 5290002, Israel; ‡ Institute of Nanotechnology and Advanced Materials (BINA), Bar-Ilan University, Ramat Gan 5290002, Israel; § Department of Chemical and Biological Physics, Weizmann Institute of Science, Rehovot 76100, Israel

**Keywords:** multilayered structures, microbubble, conducting/insulating, pattern formation, microfluidics

## Abstract

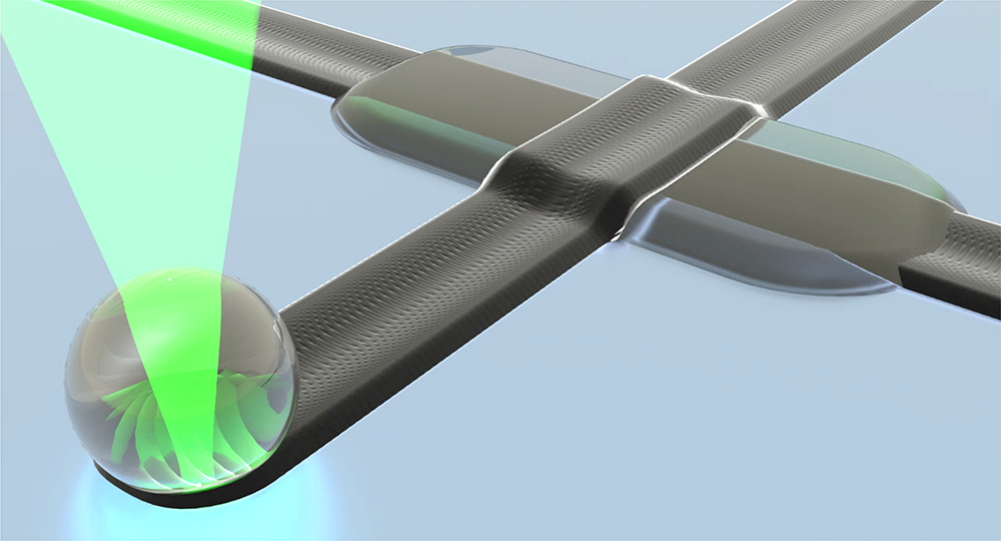

Production of multilayered microstructures composed of conducting and insulating materials is of great interest as they can be utilized as microelectronic components. Current proposed fabrication methods of these microstructures include top-down and bottom-up methods, each having their own set of drawbacks. Laser-based methods were shown to pattern various materials with micron/sub-micron resolution; however, multilayered structures demonstrating conducting/insulating/conducting properties were not yet realized. Here, we demonstrate laser printing of multilayered microstructures consisting of conducting platinum and insulating silicon oxide layers by a combination of thermally driven reactions with microbubble-assisted printing. PtCl_2_ dissolved in *N*-methyl-2-pyrrolidone (NMP) was used as a precursor to form conducting Pt layers, while tetraethyl orthosilicate dissolved in NMP formed insulating silicon oxide layers identified by Raman spectroscopy. We demonstrate control over the height of the insulating layer between ∼50 and 250 nm by varying the laser power and number of iterations. The resistivity of the silicon oxide layer at 0.5 V was 1.5 × 10^11^ Ωm. Other materials that we studied were found to be porous and prone to cracking, rendering them irrelevant as insulators. Finally, we show how microfluidics can enhance multilayered laser microprinting by quickly switching between precursors. The concepts presented here could provide new opportunities for simple fabrication of multilayered microelectronic devices.

## Introduction

Fabrication of multilayered microstructures composed of insulating and conducting materials is of great interest as they are utilized as components in various microelectronic devices such as capacitors,^1,2^ transistors,^3^ inductors,^2^ light-emitting diodes,^4^ and batteries.^4^ Other possible applications include printed circuit boards,^5^ solar cells,^6^ and medical devices.^7^ The purpose of the insulating layers is to electrically separate between conductive layers^3^ and to optimize charge balance.^8^ In addition, such layers are often deposited for protection, e.g., to avoid oxidation of the conductive layers.^5^ The thickness of insulating layers ranges between a few nanometers to several microns.^9,10^ Fabrication of conducting/insulating microstructures is currently performed mostly by photolithography methods,^11−13^ which provide layers with controlled and homogeneous thickness.^9,14^ However, similar to other top-down approaches, these methods require multiple production steps, masks, and expensive fabrication setups and are considered wasteful in terms of materials and energy consumption. Bottom-up printing methods such as ink-jet,^1,9,14^ aerosol-jet,^15,16^ and screen-printing^4^ were used to form microstructures containing conducting/insulating layers with minimal material waste.^4^ Stereolithography^17−19^ could also be adapted to form such structures. However, these methods usually employ polymers or ionic liquids as insulating layers rather than the more stable inorganic oxides, often require stabilization of the dispersions by undesired additives, and involve post-processing steps.^4^ The minimal feature size demonstrated was tens of microns, while aerosol and screen printing only provided relatively thick layers (several microns).

Light-matter interactions gained wide attention during recent decades and were shown to promote material deposition. Laser assembly from liquid precursors provides a smaller feature size than that from powders while allowing relatively simple setups, easy handling, and recycling. Many demonstrations of this approach were presented using a similar basic setup, with a surprising number of underlying mechanisms.^20^ These methods can be divided according to the source of the deposited material: preformed or locally synthesized. The study presented in this manuscript utilizes a combination of methods from both approaches: microbubble-assisted printing of preformed materials with thermally driven reactions where locally synthesized materials are deposited. Microbubble-assisted printing was shown to deposit metals,^21,22^ polymers,^23−25^ semiconductors,^24^ and organic molecules,^26^ while thermally driven reactions showed depositions of metals,^27−29^ oxides,^27,28,30−34^ organic molecules,^35^ and molecular compounds.^28^ Multilayered structures were demonstrated using thermally driven reactions,^30^ but the minimal thickness of several microns per layer renders them unsuitable for many microelectronic applications. In addition, electron transport studies on oxides were not performed; therefore, their relevance for conducting/insulating/conducting microstructures is not clear. A combination of microbubble-assisted printing with thermally driven reactions was demonstrated for assemblies of metals,^36^ oxides,^36,37^ polymers,^38^ metal–organic frameworks,^39^ and alloys.^40^ Experimental studies^36^ showed that NPs are formed in the liquid phase and carried to the microbubble base where they are deposited along with materials that are locally synthesized at the same location.

Formation of the desired microstructures is challenging as the deposition process is relatively aggressive, and the integrity of the underlying layers can be compromised. In addition, pores and cracks in the insulating layer must be avoided while maintaining minimal thickness. After examining various materials as candidates for the insulating layer, we found that tetraethyl orthosilicate (TEOS) can be used to form silicon oxide layers, which can withstand microprinting of additional layers on top of them while maintaining insulating properties. We demonstrate control over the height of the insulating layer between about 50 and 250 nm by increasing the laser power or the number of layers. Moreover, we show that a microfluidic channel can be utilized to quickly switch between media, enabling rapid production of multilayered microstructures. These new approaches could therefore provide new opportunities for various applications such as multilayered microelectronic devices and microcapacitors.

## Results and Discussion

**Figure 1:**
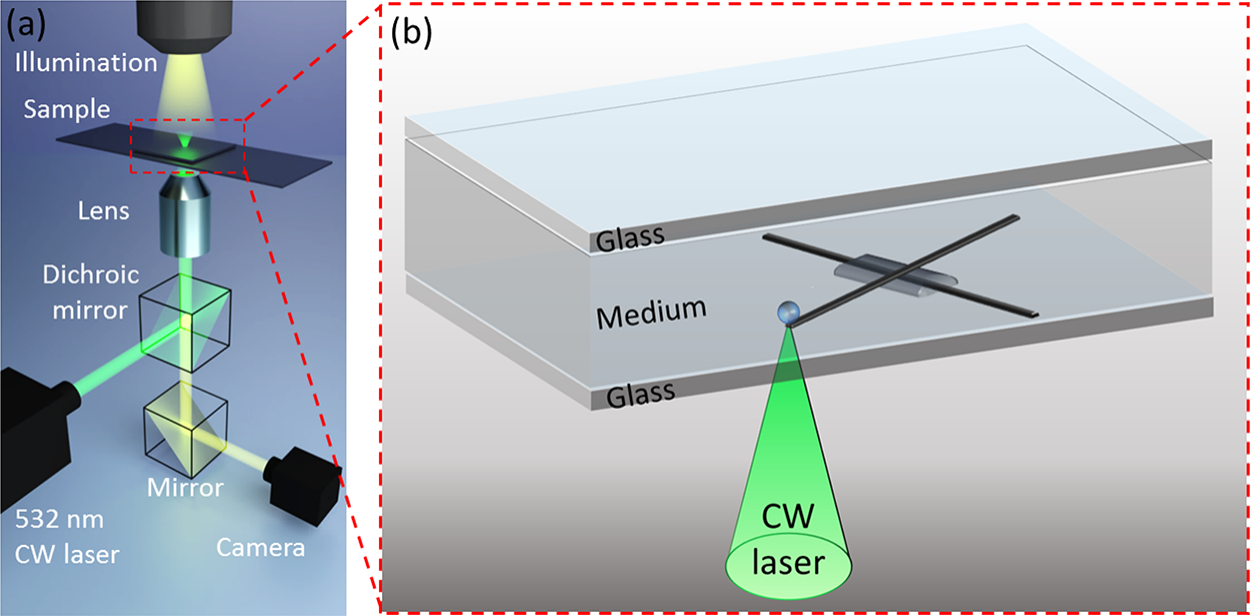
Illustrations of (a) optical setup and (b) sample geometry. Multilayered micropatterns are fabricated between two glass slides filled with precursors by moving the microscope stage relative to the sample.

A 532 nm continuous wave (CW) laser was focused on the liquid medium (Figure 1a) containing the required precursors inserted between two glass slides (Figure 1b). PtCl_2_ dissolved in *N*-methyl-2-pyrrolidone (NMP) was used to form conducting Pt layers, while TEOS dissolved in NMP containing small amounts of aqueous NaOH was found to form insulating silicon oxide layers by condensation polymerization (Figure S1). The microscope stage was computer-controlled, and the experiments were recorded using a CMOS camera. To form patterns, unless otherwise stated, the microscope stage was moved along a predetermined path at 100 μm/s for Pt deposition and 400 μm/s for silicon oxide deposition with a laser illuminating power of 14 mW. Laser modulation was used to achieve continuous depositions (see more details in the Experimental Section).

**Figure 2:**
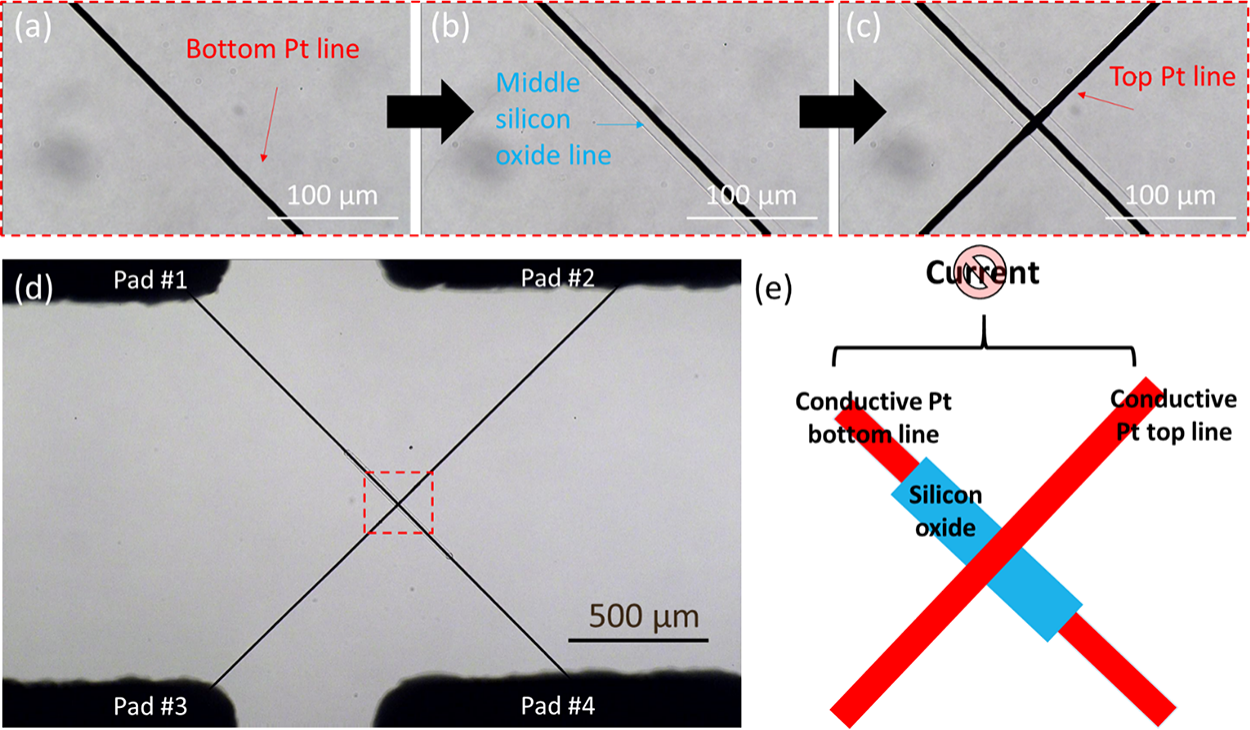
(a–c) Bright-field microscopy images showing the steps for fabricating conducting/insulating/conducting microstructures: (a) microprinting the bottom Pt conducting line, (b) silicon oxide layer, and (c) top Pt line. (d) Bright-field microscopy zoomed-out image and (e) illustration presenting the scheme for electrical measurements.

**Figure 3:**
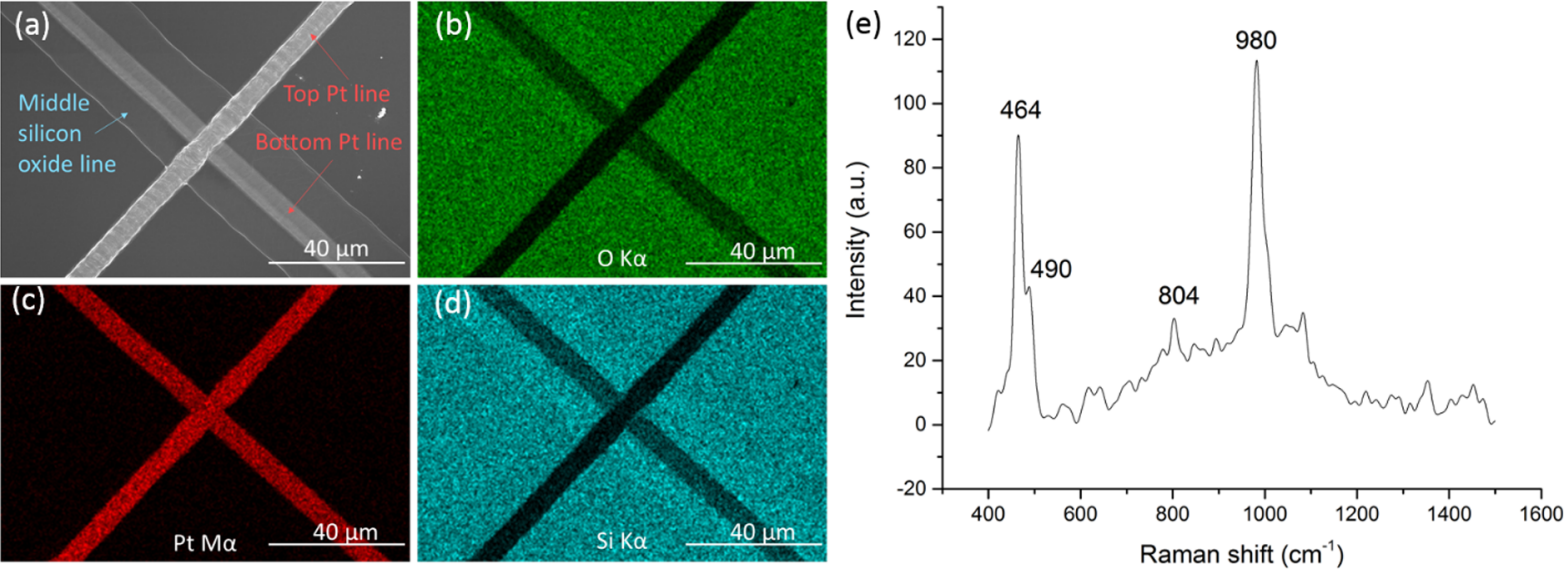
(a) Scanning electron microscopy image of the fabricated microstructure. (b–d) EDS mapping demonstrating the presence of O, Pt, and Si in the appropriate layers, respectively. (e) Raman spectrum (using a 785 nm laser) of the microprinted silicon oxide layer on gold.

To examine the electrical transport properties of the insulating layer, conducting/insulating/conducting multilayered microstructures were formed (Figure 2). A cover slide with four sputtered gold pads was used as the substrate. A Pt line (Figure 2a) was deposited, connecting two of the gold pads (Figure 2d), and an insulating silicon oxide layer was selectively deposited on top of the Pt line only in the area later used to form an electrical junction (Figure 2b). To achieve good electrical isolation, three repetitions of the silicon oxide deposition process were performed, and the line was deliberately wider than the underlying Pt line, fully covering even its edges. Finally, another Pt line was printed between two other gold pads, perpendicular to the previously printed lines. This line forms an electrical junction with the silicon oxide in between the two Pt lines (Figure 2d,e). High-resolution scanning electron microscopy (HR-SEM) images (Figure 3a) show that all three layers seem to be continuous and that the middle layer fully covers the bottom Pt line. EDS mapping (Figure 3b–d) is consistent with the expected chemical elements. The entire process is shown in Video S1 in the Supporting Information. A microbubble is clearly visualized throughout the printing process. Its role in material deposition is discussed below.

We performed Raman measurements (Figure 3e) that agreed with previously reported spectra of silicon oxides. The peak at 464 cm^–1^ corresponds to the silane network binding (Si–O–Si stretching-bending),^41,42^ while 490 cm^–1^ is associated with vibrational modes of tetracyclosiloxane rings.^43−45^ The peak at 804 cm^–1^ corresponds to Si–O antisymmetric stretching^46^ or Si–O–Si bending.^47^ The large peak at 980 cm^–1^ is assigned to Si–OH stretch vibrations.^45,48^ The 464 cm^–1^ peak could be indicative of a crystalline structure, and as for amorphous silica, there is a significantly broader peak around the same location.^42^ Both amorphous and crystalline structures might be present, and a dedicated study should be performed to clarify the crystallinity ratio. There is no significant signal around 1300–1400 cm^–1^ (D band) typical of carbon-based materials, suggesting that residual carbon is minimal.

**Figure 4:**
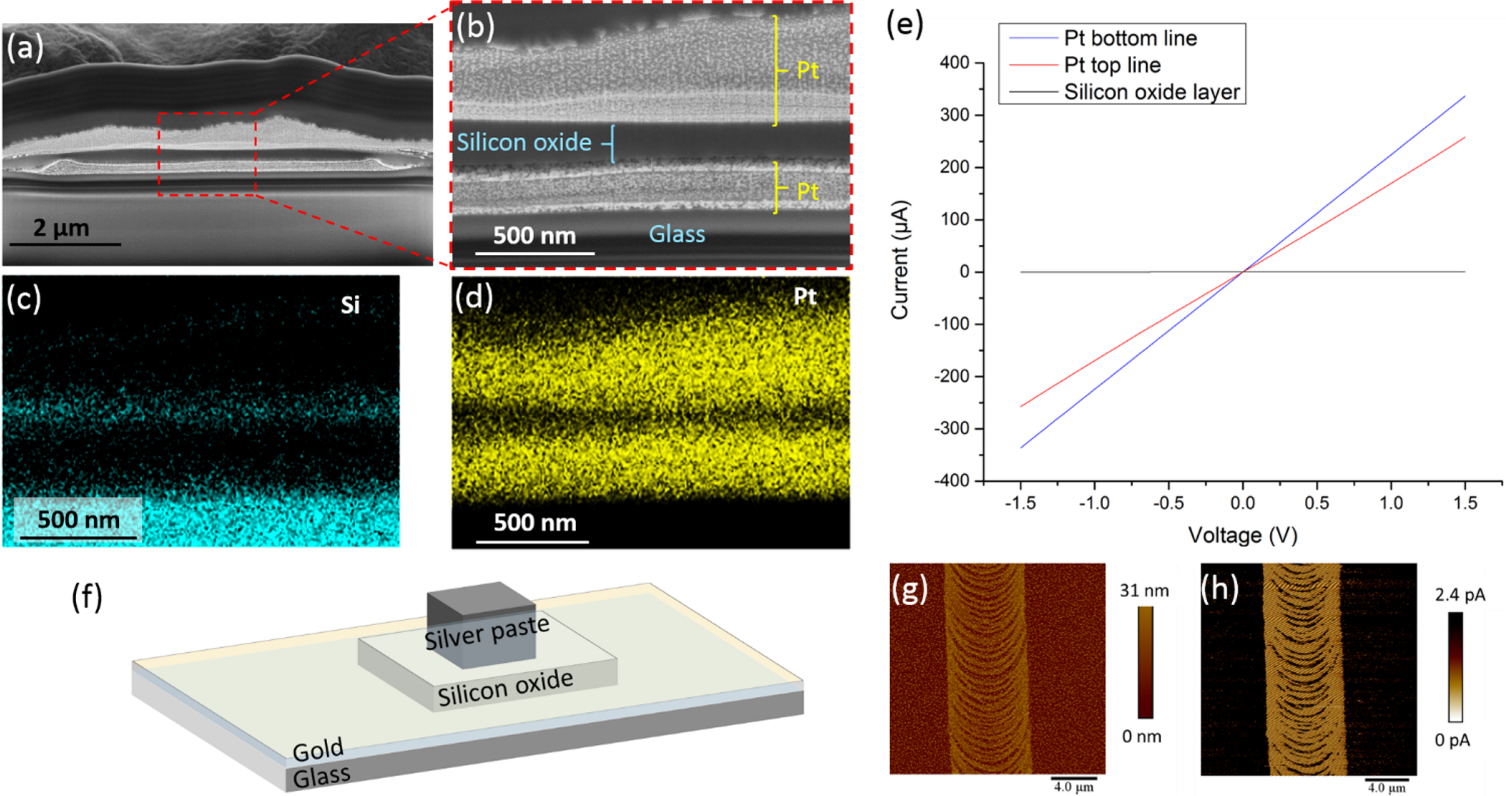
(a, b) HR-SEM images and (c, d) EDS mappings of Si and Pt of the FIB cross-sectioned multilayered microstructure. (e) Typical *I*–*V* curves of the three different contact combinations of the layered junction. Measurements along the bottom (pads #1 and #4) and top (pads #2 and #3) Pt lines indicate that they are conductive, while measurements between the top and bottom lines (pads #1 and #2 or #3 and #4) indicate high resistivity of silicon oxide. (f) Illustration of the Ag/silicon oxide/gold multilayered structure fabricated for evaluating the resistivity of silicon oxide. (g) Atomic force microscopy topography image and (h) TUNA measurements of thin (down to ∼10 nm) silicon oxide layers, yielding currents comparable to the background noise, indicating high resistivity.

For investigating the sub-micron structure of the fabricated layers, we performed cross-sectioning by focused ion beam (FIB). HR-SEM imaging (Figure 4a,b) along with EDS mapping of Si and Pt (Figure 4c,d) reveals the presence of the three layers. The thickness of the silicon oxide layer is ∼170 nm. Moreover, the images show that the relatively aggressive deposition process of the second and third layers did not compromise the integrity of the underlying layers. The insulating layer shows neither pores nor cracks.

Electrical measurements show that the Pt lines are conductive and show a resistivity of 3.2 ± 0.6 × 10^–6^ Ωm. This value is slightly better than previously reported state-of-the-art values for laser-based microprinted Pt (4.2 ± 0.5 × 10^–6^ Ωm).^49^ To characterize the nanostructure of Pt depositions, TEM lamellae were formed using FIB. As shown in Figure S2a, the deposits are composed of fused nanocrystals with clear atomic planes. Selected area diffraction pattern (SADP) measurements (Figure S2b) show *d*-spacing associated with face-centered cubic (fcc) platinum. EDS measurements of Pt lines (Figure S2c) did not show evidence of residual elements (such as Cl or C).

Figure 4e shows typical *I*–*V* curves of the three different contact combinations of the layered junction. Measurements along the bottom (pads #1 and #4) and top (pads #2 and #3) Pt lines indicate that both lines are conductive, while measurements between the two lines (pads #1 and #2 or #3 and #4) show high resistance. For evaluating the resistivity of the silicon oxide, a bigger contact area is required. We therefore prepared a layered structure by laser microprinting of silicon oxide on top of gold evaporated on a glass substrate. Silver paste was applied on the silicon oxide layer, achieving a Ag/silicon-oxide/gold multilayered structure (Figure 4f). The resistivity of the silicon oxide layer was found to be 1.5 ± 0.5 × 10^11^ Ωm between 0 and 0.5 V. This value is sufficient for various applications; however, it is about 2 orders of magnitude lower than previously reported values for silicon oxide,^50^ possibly due to the incorporation of precursors in nanopores. In addition, tunneling atomic force microscopy (TUNA) measurements were performed on the thinnest silicon oxide layers that we were able to prepare on an evaporated gold layer (Figure 4g,h) by significantly increasing the stage velocity (to 10 mm/s, the fastest our stage can move). Note that these layers are the only non-continuous ones in this study and are atypical compared to the other layers of silicon oxide that provide uniform coverage. We found regions of silicon oxide as thin as ∼10 nm; however, even these layers were too insulating to allow quantitative analysis. TUNA measurements showed currents comparable to the background noise, indicating high resistivity of the silicon oxide layers.

Prior to our findings on microprinting of TEOS presented above, we examined various materials that were expected to form suitable insulating layers. A list of the materials is provided in the Supporting Information. While we have not performed an in-depth study on each material, here, we shortly hypothesize why they failed to form appropriate insulating deposits. The materials can be divided into two categories: preformed NPs and inorganic metal ion precursors. Deposition of electrically insulating NPs such as SiO_2_, ZrO_2_, and TiO_2_ by microbubble-assisted printing did not form continuous layers due to poor adhesion between the NPs. Addition of binders (such as polyvinylidene difluoride) allowed continuous layered formation; however, the deposits were still too porous to act as effective insulating layers. While inorganic metal ion precursors could form oxide layers, these proved to be highly porous or have cracks, rendering them inappropriate for electrical insulation (Figure S3). Silicon oxide layers formed from TEOS did not show cracking, and pores were not visible in HR-SEM. This might be attributed to the organic nature of the silicon oxide precursor (TEOS) in contrast with the inorganic precursors of the other oxides used. Although nanopores were reported for such layers,^51^ we hypothesize that they are small enough to prevent penetration of the top metallic layer. However, further investigation beyond the scope of this paper is required to validate this hypothesis.

**Figure 5:**
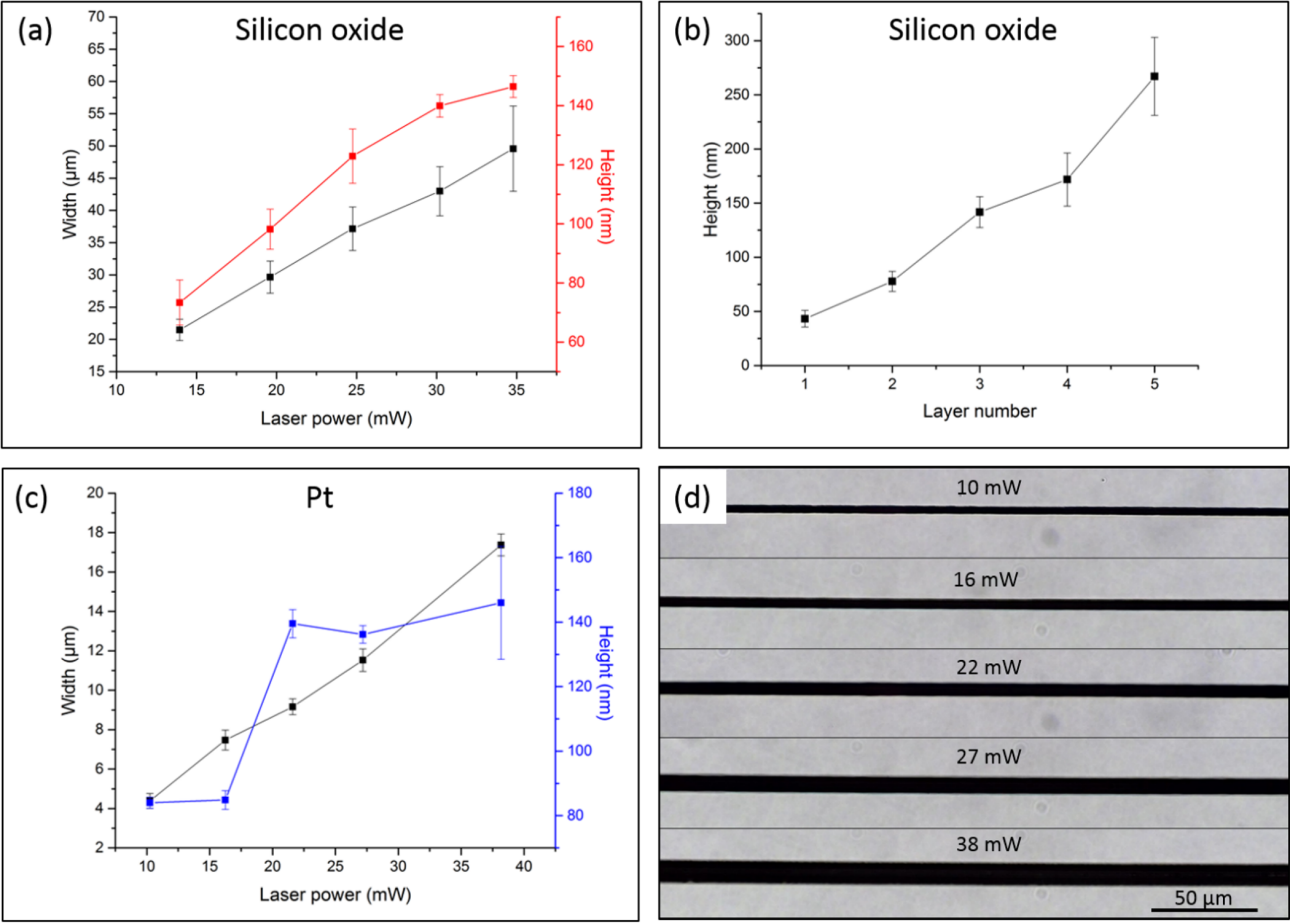
(a) Width and height of silicon oxide lines as a function of the laser power and (b) layer number. (c) Width and height of Pt lines as a function of the laser power and (d) corresponding bright-field microscopy images.

This laser-based microprinting method also enables control over the width and height of fabricated conducting and insulating layers. For silicon oxide (Figure 5a), as the power varied from 14 to 35 mW, the obtained deposition width increased from about 21 to 50 μm. In addition, the height of the deposits increased with the laser power from about 70 to 150 nm. Similar results were found for Pt layers (Figure 5c,d). Finally, repeating the deposition procedure several times (silicon oxide deposition on a printed Pt line) by moving the laser back and forth also increased the layer height (Figure 5b).

**Figure 6:**
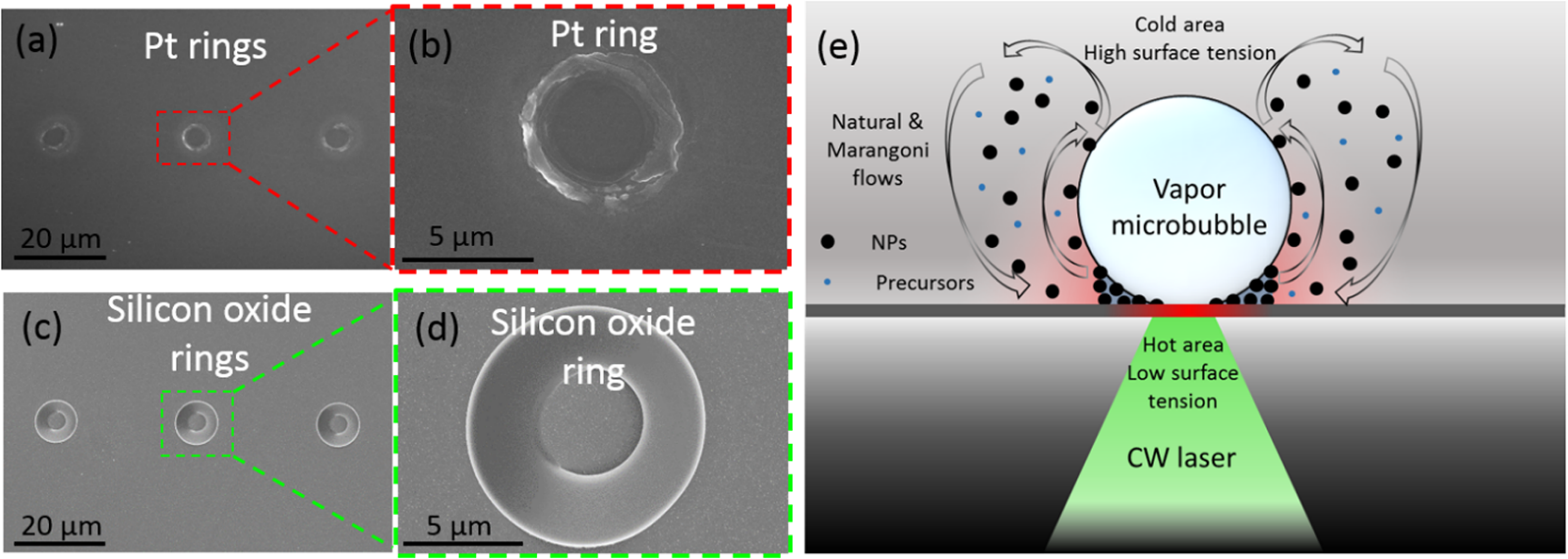
SEM images of (a, b) Pt and (c, d) silicon oxide demonstrating ring-shaped patterns indicative of material deposition at the contact area of a microbubble with the underlying surface. (e) Illustration of the deposition mechanism by thermally driven reactions with microbubble-assisted printing. NPs are first created followed by a microbubble from the vapors of the liquid medium. Convection flows at the vicinity of the microbubble carry NPs, and some of them are transferred to the microbubble/substrate interface where they are pinned. Thermal reactions also occur at the microbubble/substrate interface and form products that fill the gaps between the deposited NPs (gray area).

The microbubbles that are seen during the deposition process (Video S1) are crucial for material deposition, and we have not attained material deposition that was not accompanied by microbubble formation. Additionally, when the shutter was set to allow a laser illumination of 50 ms, ring-shaped patterns were formed (Figure 6a–d), indicating material deposition of Pt and silicon oxide at the contact area of the microbubble with the underlying surface. Based on a previous study,^36^ we suggest that the mechanism combines deposition by thermally driven reactions with microbubble-assisted printing (Figure 6e). NPs of Pt or silicon oxide are first created, and heat generated by laser light absorption increases the vapor pressure of the liquid until a microbubble is formed. When microprinting Pt, the platinum NPs absorb the 532 nm photons, while for silicon oxide, which is transparent, the substrate (previously deposited Pt) absorbs the photon energy. Convection flows at the vicinity of the microbubble carry NPs, and some of them are transferred to the microbubble/substrate interface where they are pinned. Thermal reactions also occur at the microbubble/substrate interface and form products that fill the gaps between deposited NPs (gray area in Figure 6e).^36^ Moving the microscope stage relative to the fixed focused laser results in propagation of the microbubble to a new location.^52,53^ Therefore, while the deposits are ring-shaped around the base of the microbubble, continuously moving the stage provides dense depositions of Pt and silicon oxide. We note that laser modulation was used to allow formation of continuous depositions. Previous studies have shown that modulation allows better control over the size of the microbubble and prevents it from being pinned to the deposited material while moving.^21,36^ The increase in both the width and height with the laser power shown in Figure 5a,c,d can be rationalized as follows. The increase in absorbed light creates a bigger microbubble, leading to wider lines. The greater amount of absorbed energy also promotes excess material synthesis and deposition, which pile higher.

**Figure 7:**
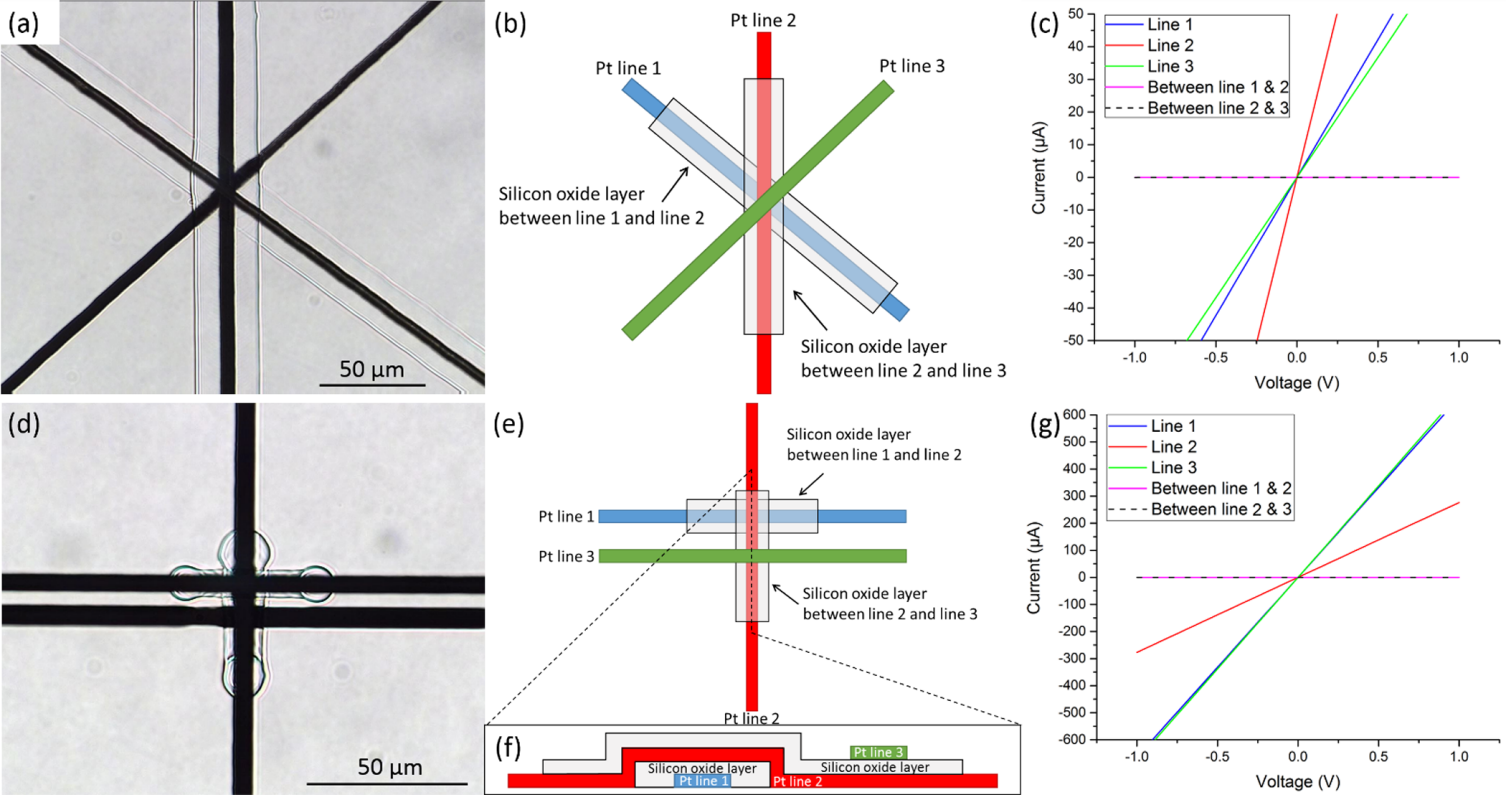
(a) Bright-field microscopy image, (b) illustration, and (c) corresponding *I*–*V* measurements of a five-layered junction. (d) Bright-field microscopy image, (e, f) illustrations (not to scale), and (g) corresponding *I*–*V* measurements of two interwoven junctions with an ∼5 micron separation.

Junctions with higher complexity levels could also be attained using our approach. Figure 7a demonstrates a five-layered junction consisting of three Pt lines separated by two silicon oxide layers, as illustrated in Figure 7b. *I*–*V* measurements (Figure 7c) indicate conductance of the Pt lines, while the high resistivity of silicon oxide layers is maintained. Two interwoven junctions with an ∼5 micron separation are shown and illustrated in Figure 7d–f. Corresponding *I*–*V* curves are presented in Figure 7g. Deposition of narrower silicon oxide layers (compared to what has been shown above) was achieved using a laser power of 8.5 mW and a stage velocity of 600 μm/s. We note that the line spacing of conducting lines can be further reduced (Figure S4). Additionally, junctions could be formed by a combination of more than one metal as the conducting element. This was demonstrated by forming a junction of Au/silicon oxide/Pt (Figure S5).

**Figure 8:**
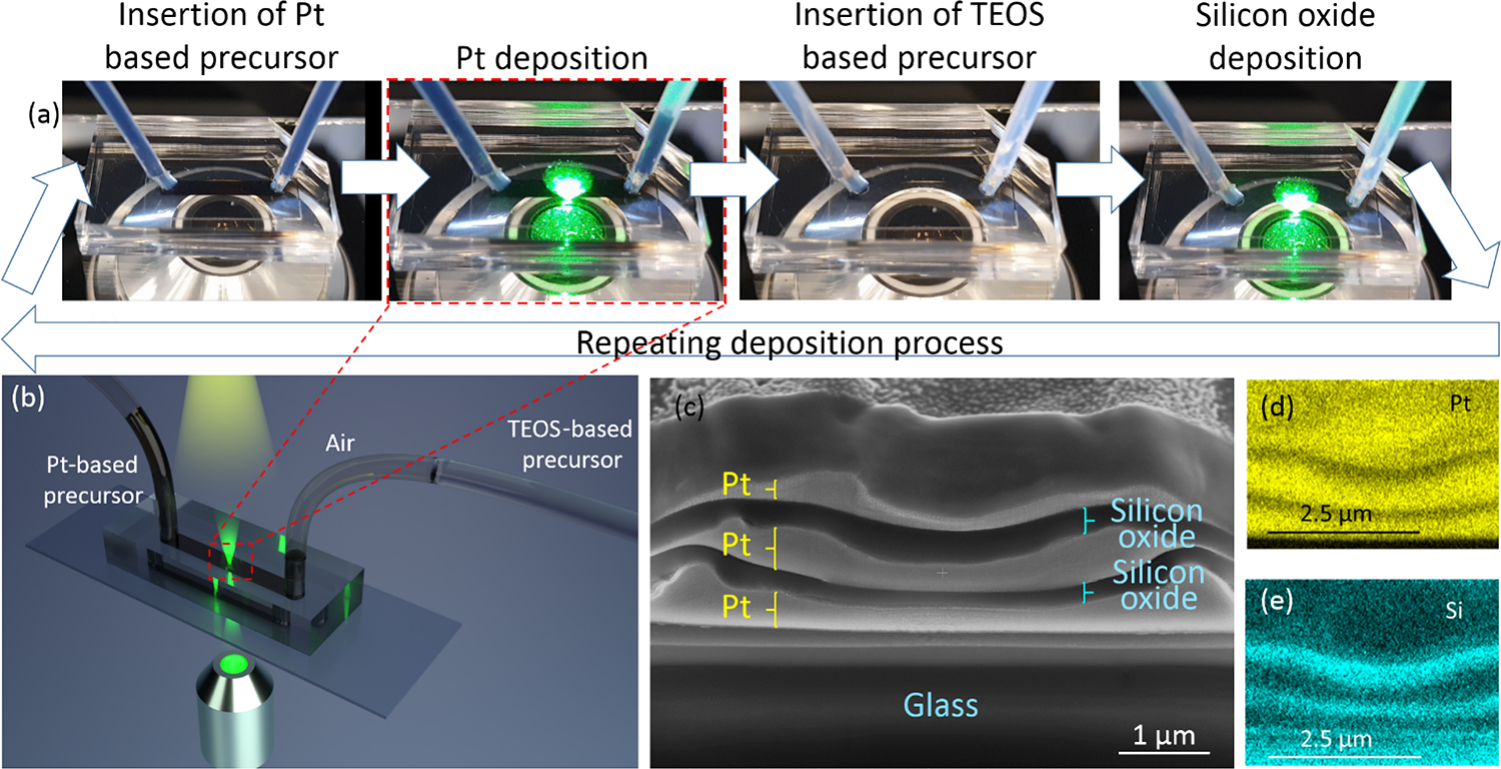
Microfluidic configuration utilized for multilayered laser microprinting, allowing a faster and more reliable production of conducting/insulating/conducting microstructures. (a) Images of the printing process displaying the steps for printing Pt and silicon oxide layers. This process can be repeated several times. (b) Illustration of the combined laser printing and microfluidic setup. A microfluidic channel is connected at both edges by tubes. Air is used as a separator between the two precursors inserted into the tubes. A pressure-based microfluidic flow controller is used to select which precursor is placed on the glass substrate and deposited by laser microprinting. (c) HR-SEM image and (d, e) EDS mapping of the FIB cross-sectioned multilayered microstructure fabricated using the microfluidic channel.

Finally, we introduce a new concept where microfluidics is utilized to enhance the capabilities of multilayered laser microprinting. The study presented above requires manually removing the liquid precursors of one component before applying the second component. This is a time-consuming and error-prone process, which makes this approach unpractical if many layers are required. We therefore suggest using pressure-controlled microfluidic channels to quickly switch between precursors (Figure 8a), allowing faster production of conducting/insulating/conducting multilayered microstructures. A polydimethylsiloxane (PDMS) microchannel (2 mm wide × 100 μm high) was formed by standard lithographic methods and was attached to a glass slide on which our microprinting took place. The microchannel was connected at both edges by polytetrafluoroethylene (PTFE) tubes. The Pt-based precursor was inserted into one of the tubes, while the TEOS-based precursor was inserted into the other. Air was deliberately trapped in between the two precursors, functioning as a separator. Both tubes were connected to a microfluidic pressure-based flow controller. This configuration (Figure 8b) allowed us to select which precursor would be placed inside the microfluidic channel where laser microprinting is performed. We repeatedly switched between precursors (Figure 8a and Video S2) to form multilayered structures visualized in a HR-SEM image (Figure 8c). Figure 8d and Figure 8e show EDS mappings of Pt and Si, respectively. Switching between precursors in this setup took approximately 10 s, a major improvement over previous manual methods. However, we did not optimize this process. Reducing the amount of air and increasing the flow velocity could lead to substantially lower transition times. Microprinting multiple elements on larger areas could conceptually be achieved utilizing microfluidics by increasing the microchannel width, using several microchannels, or shifting the microchannel from region to region.

## Conclusions

In conclusion, laser-based microprinting of layered conducting/insulating/conducting microstructures was demonstrated with silicon oxide in between platinum conducting layers. Several configurations were shown, and cross sections of the microstructures were visualized by HR-SEM and mapped with EDS. Electrical transport measurements were performed to examine the properties of the insulating layer, yielding a sufficiently high resistivity of 1.5 × 10^11^ Ωm at 0.5 V. Control over the width and height of the insulating layer was demonstrated by increasing the laser power and number of iterations. Finally, microfluidics were utilized to eliminate the manual, time-consuming, and error-prone process of switching between precursors. This novel approach could prove to be beneficial for fabrication of various multilayered microelectronic devices.

## Experimental Section

### Sample Preparation

Pt was chosen due to its good adhesion to the substrate. For the preparation of the 3 wt % Pt-based precursor, 30 mg of Pt(II)Cl_2_ and 0.97 g of dry NMP (Acros Organics) were added to a glass vial and stirred on a tube roller at room temperature for a week. The obtained Pt-based precursor was filtered using a syringe filter (polytetrafluoroethylene, Membrane Solutions) with a 0.4 μm pore size before microprinting. Note that NMP was chosen due to its relatively high boiling point (202 °C), which is preferable for both microbubble-assisted printing and thermal synthesis.^23^ NMP also promotes the thermal reduction process of Pt ions to a stable dispersion of Pt NPs. The detailed chemical mechanism of metal ion reduction by NMP is not yet fully understood.^54^

The TEOS precursor solution was prepared by adding 725 μL of NMP to 250 μL of TEOS (Sigma-Aldrich) followed by mixing with a vortex shaker. Then, 25 μL of 2 M NaOH (Thermo Fisher Scientific) in deionized water was slowly added while stirring. The obtained precursor was passed through a 0.4 μm pore size syringe filter before microprinting.

Sample preparation consisted of cleaning microscope cover glass slides (0.17 mm thick) with isopropanol followed by drying. A cavity was formed by placing one of the cover slides as a spacer in between two slides. Using a pipette, 50 μL of the Pt- or TEOS-based precursors was inserted by capillary force into the cavity. A glass substrate sputtered with 5 nm Cr followed by 100 nm gold was used for conductivity and resistivity measurements as well as results shown in Figure 5a and Figure 6a–d.

### Optical Setup

The optical setup (Figure 1a) consisted of a CW laser beam (532 nm, Verdi G-Serie, Coherent) integrated with a bright-field inverted microscope (Nikon Eclipse Ti-U) and focused by a 40× objective lens (0.6 NA, Nikon). The laser power, as measured after the objective lens with a power meter (PM100, Thorlabs) and without applying the modulation, was 14 mW unless otherwise stated. The stage of the microscope was computer-controlled, and the experiments were recorded using a CMOS camera (DPCAM 6CHDMI, DeltaPix). To produce the microstructures, the microscope stage was moved with a stage velocity of 100 μm/s for Pt and 400 μm/s for silicon oxide unless stated otherwise. Laser modulation for Pt patterns was performed by an optical chopper (Thorlabs) with a frequency of 3 kHz and a duty cycle of 50%. The ring-shaped patterns of both Pt and silicon oxide (Figure 6a–d) were formed by limiting the exposure time of the laser beam to 50 ms by a mechanical shutter (SH1, Thorlabs).

### Microfluidic Channel Fabrication and Setup

A mixture of dimethylsiloxane and cross-linking agents (Sylgard 184) was poured on a mold containing the positive relief of the channel’s layout prepared using conventional photolithography. Curing was obtained at 80 °C for 2 h. After curing, the PDMS layer was detached from the mold, forming an open microchannel. Holes were punched in PDMS to allow tube connection. A clean glass slide (1 mm thick) was attached to the microchannel followed by 12 h at 80 °C. Usually, plasma treatment is preferred for the PDMS microchannel and glass to strengthen the bond between them and permanently attach them together. However, as access to the glass substrate is required after micropatterning for imaging, plasma treatment was avoided. The final microfluidic device contains a single channel, 2 mm wide, 100 μm high, and 10–30 mm long. The microchannel was connected at both edges by PTFE tubes. The Pt- and TEOS-based precursors were inserted into the tubes with air functioning as a separator deliberately trapped in between. Both tubes were connected to a microfluidic pressure-based flow controller (Elveflow OB1 MK3 system).

### Characterization Methods

HR-SEM imaging, FIB cross-sectioning, and EDS measurements were performed using a Helios 5 UC dual-beam system (Thermo Fisher Scientific). The nanostructure was considered with transmission electron microscopy on JEOL-2010 HR-TEM using an accelerating voltage of 200 kV, and elemental analysis was conducted by energy-dispersive X-ray spectroscopy (EDS) with a spot size of 35 nm. SADP measurements were performed with an accelerating voltage of 200 kV and a spot size of 400 nm.

Raman measurements were performed using a customized setup based on a Zeiss Axio Vario Scope A1 microscope equipped with a 785 nm laser with a power of 50 mW. Grating (600 L/mm) and a 50× objective lens were used. Resistance measurements were performed at a SUSS MicroTec probe station. Local electrical conductivity was measured using a MultiMode AFM system (Bruker AXS) equipped with a highly sensitive current detector (TUNA module) with a set point of 0.47 V. All images were obtained using TUNA mode with a SCM-PIC-V2 (Bruker) Pt/Ir-coated silicon probe (spring constant of 0.2 N/m). The resonance frequency of the cantilever was approximately 13 kHz (in air). The measurements were performed under environmental conditions. The images were captured in the retrace direction with a scan rate of 0.5 Hz. The resolution of the images was 512 samples per line. Image processing was performed using Nanoscope Analysis software. Prior to analysis, the “flatting” and “planefit” functions were applied to each image.

Widths, heights, and cross-sectional areas of deposited Pt layers were obtained by an optical profilometer (LEXT, OLS4100, Olympus), while for silicon oxide layers, a stylus profilometer (Veeco Dektak 150 system) was used. Measurements (Figure 5a–c) were performed on three samples prepared separately, each containing at least 10 lines. Reported heights were calculated by dividing the cross-sectional area with the width (providing an average height). Only for silicon oxide deposited on a Pt line (and not on a flat surface), the height was determined as the difference between maximum heights. Conductance of deposited Pt lines (15 separate samples) was measured by a Keithley 2400 sourcemeter equipped with a home-built four-probe station.

## Supporting Information Available

Synthesis scheme of TEOS to form silicon oxide; HR-TEM image, selected area diffraction pattern measurements and EDS measurements of lamella from a deposited Pt line; SEM images of attempts to form insulating layers from Al-based ionic solution; Pt micro-printed lines with reduced spacing; bright-field microscopy image, I-V measurements and EDS mapping of an Au/silicon-oxide/Pt junction (PDF)
Movie showing microprinting of a multilayered junction (MP4)
Movie showing fabrication of a multilayered microstructure inside a microfluidic channel (MP4)
