# Supplementary material for: Laser Printing of Multilayered Alternately Conducting and Insulating Microstructures
Source: ACS Appl Mater Interfaces. 2021 Jul 23;13(30):36416–25. doi: 10.1021/acsami.1c06204 (PMC8397236; doi:10.1021/acsami.1c06204)
Supplement: Supplementary file 1 — am1c06204_si_001.pdf [file am1c06204_si_001.pdf]

Supporting Information

Laser printing of multi-layered alternately conducting and insulating micro-structures

Eitan Edri,[1,2] Nina Armon,[1,2] Ehud Greenberg,[1,2] Shlomit Moshe-Tsurel,[1,2] Danielle Lubotzky,[1,2] Tommaso Salzillo,[3] Ilana Perelshtein,[2] Maria Tkachev,[2] Olga Girshevitz[2] and Hagay Shpaisman,[1,2]*

[1] Department of Chemistry, Bar-Ilan University, Ramat Gan 5290002, Israel

[2] Institute of Nanotechnology and Advanced Materials (BINA), Bar-Ilan University, Ramat Gan 5290002, Israel

[3] Department of Chemical and Biological Physics, Weizmann Institute of Science, Rehovot 76100, Israel

*E-mail: hagay.shpaisman@biu.ac.il

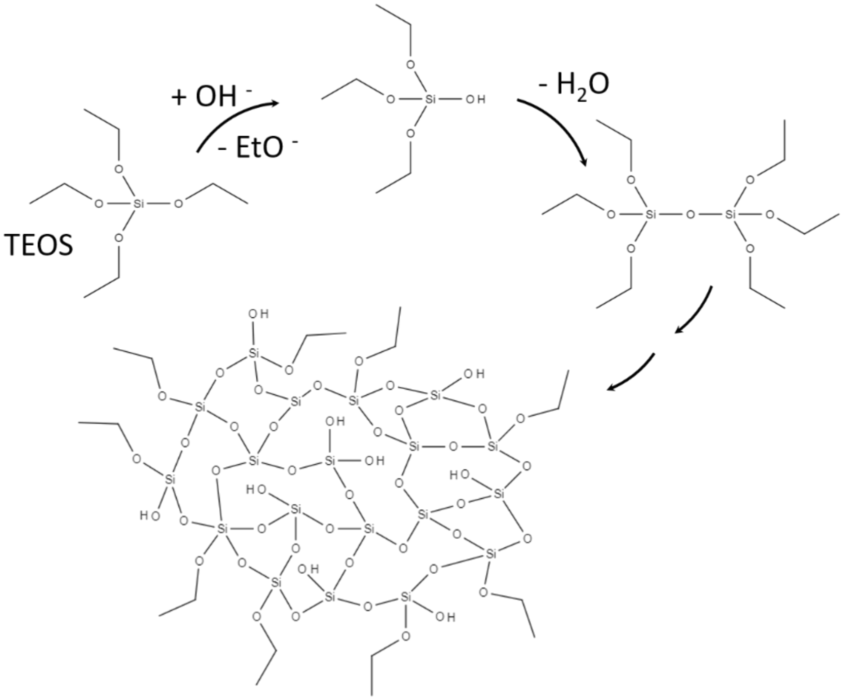

Figure S1. Synthesis scheme showing condensation polymerization of TEOS to form silicon oxide.

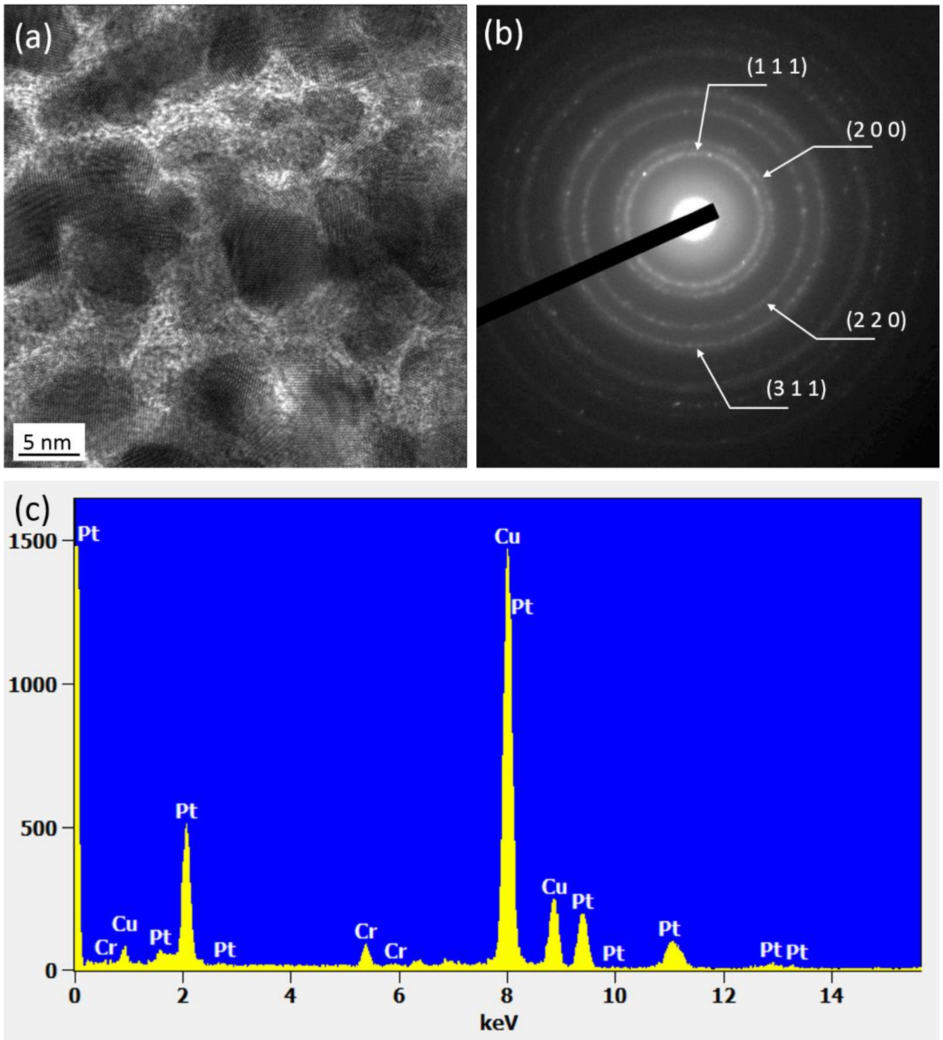

Figure S2. (a) HR-TEM image of prepared lamella from a deposited Pt line. (b) Selected area diffraction pattern measurements showing d-spacing of 2.25 Å, 1.90 Å, 1.35 Å and 1.13 Å which are in good correlation with (1 1 1), (2 0 0), (2 2 0) and (3 1 1) planes of fcc Pt, respectively, as reported by the joint committee on powder diffraction standards (JCPDS) file no: 01-087-0646. (c) EDS measurements of the Pt lamella. We note that the Cu and Cr signals arise from the TEM grid and column.

Micro-printing of other possible insulators:

Prior to our findings on micro-printing of insulating silicon oxide, we examined various materials that were expected to form suitable insulating layers. However, the materials listed below did not show reasonable insulating properties.

Dispersions of NPs:

Dispersions of 0.5–1 wt% of SiO_2 (7 nm), ZrO_2 (50 nm) and TiO_2 (50, 100 nm) were prepared in three solvents – water, diethylene glycol dibutyl ether (DB, Alfa Aesar), and N-methyl-2-pyrrolidone (NMP). In all cases, deposition by micro-bubble assisted printing did not form continuous layers due to poor adhesion between the NPs. Addition of 0.1 wt% of polyvinylidene difluoride to serve as a binder allowed continuous layered formation, however the deposits were too porous to act as effective insulating layers.

Solutions containing inorganic precursors:

Solutions of 3 wt% of $AlCl_3$ (Fluka) were prepared in DB, NMP, and water. Depositions by a combination of thermally driven reactions with micro-bubble assisted printing on top of Pt lines resulted in porous layers (**Figure S2a**). Addition of 0.5 wt% polyvinyl alcohol (PVOH, Aldrich) showed no improvement (**Figure S2b**). Solutions of 3 wt% $Al(NO_3)_3$ (Merck) in a 0.5 M NaOH solution with and without 0.5 wt% PVOH were also prepared; depositions on Pt lines by thermally driven reactions with micro-bubble assisted printing formed layers with cracks visible in SEM (**Figure S2c**,**d**). These layers were found to be non-insulating. Depositions from solutions of 15 wt% of $TiCl_3$ in 10% HCl (Merck) and 3 wt% of $(NH_4)_6Mo_7O_{24}$ (Fisher Scientific) in water also failed to form insulating layers.

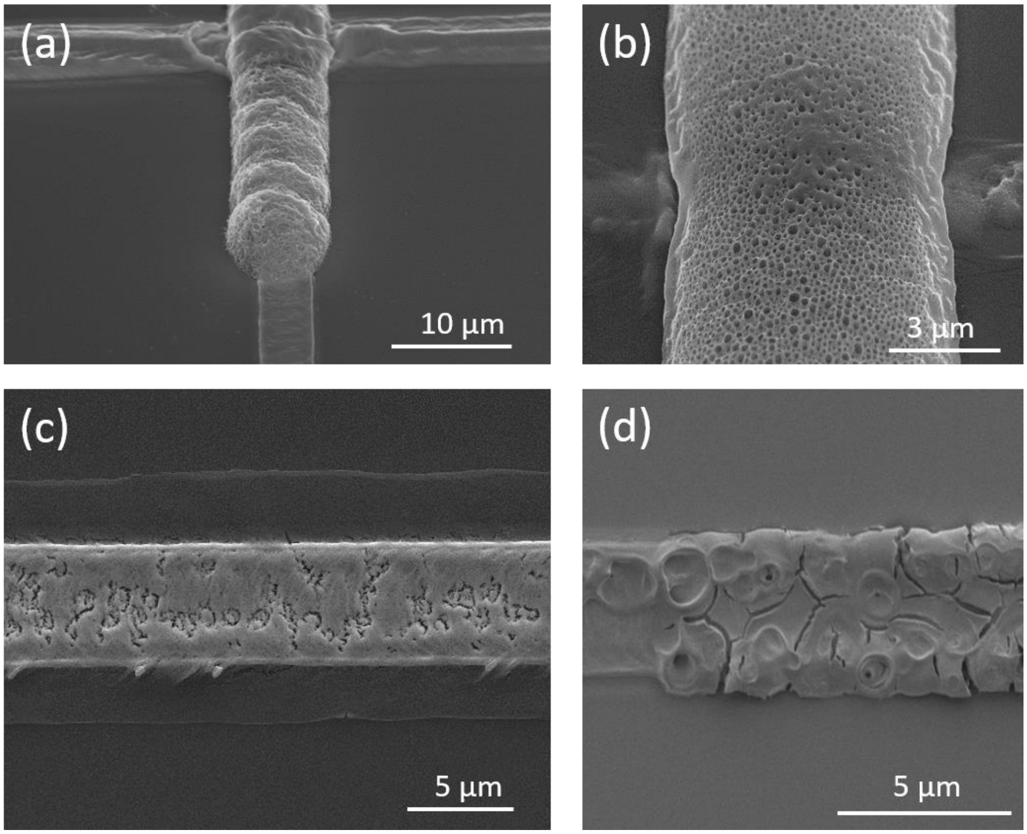

Figure S3. SEM images of depositions fabricated by thermally driven reactions with micro-bubble assisted printing on top of a Pt line that failed to form an insulating layer. The precursors used were: (a) 3 wt% $AlCl_3$ in water, (b) 3 wt% $AlCl_3$ in water with 0.5 wt% polyvinyl alcohol, (c) 3 wt% $Al(NO_3)_3$ in a 0.5M NaOH solution and (d) 3 wt% $Al(NO_3)_3$ with 0.5 wt% PVOH.

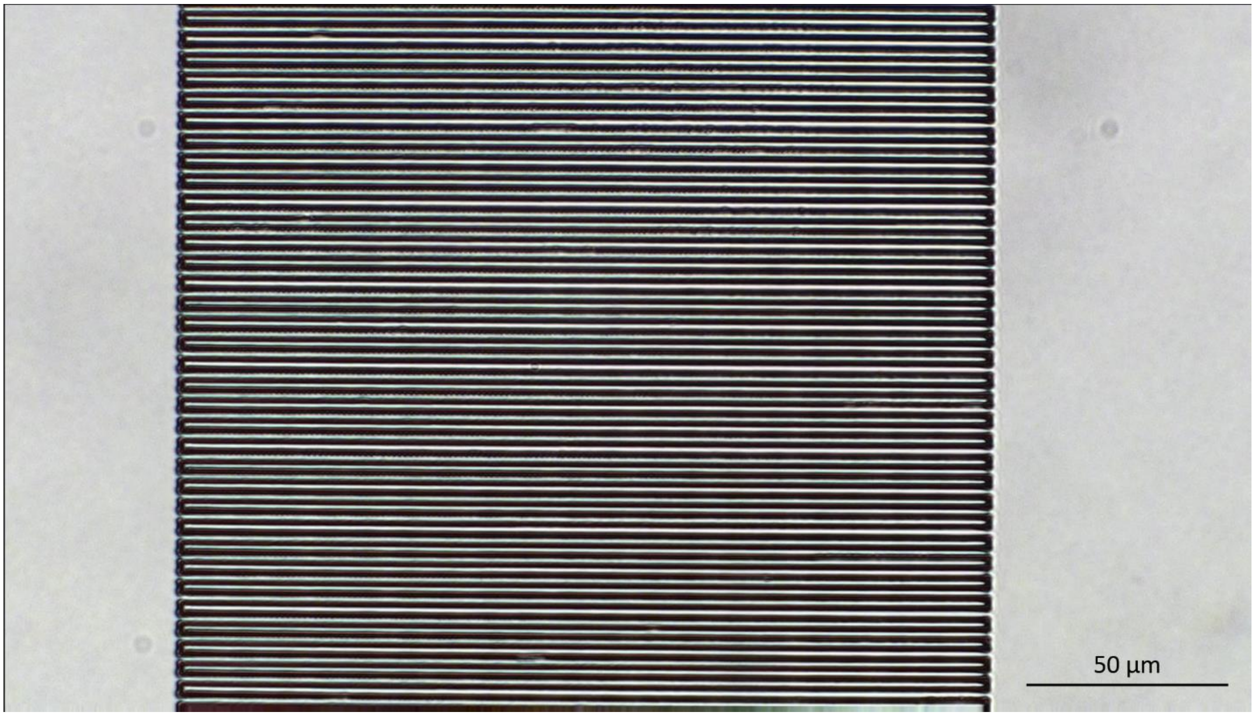

Figure S4. Pt micro-printed lines with reduced spacing. Each line is ~1.5 microns wide with a spacing of ~1 micron. Parameters used: stage velocity of 60 µm/s and laser power of 5mW.

Micro-printing of an Au/silicon-oxide/Pt junction

Au precursor solution was prepared by mixing 400 µl of a 0.125 M NaOH aqueous solution with 100 µl of 0.1 M $HAuCl_4$ aqueous solution. Deposition of the bottom metal Au layers was performed using laser power of 54 mW, stage velocity of 60 µm/s with laser modulation of 3 kHz. Silicon oxide and Pt layers were micro-printed with the same parameters reported in the experimental section. I-V measurements (**Figure S5b**) indicate conductance of Pt and Au lines while high resistivity of the silicon oxide layer is maintained.

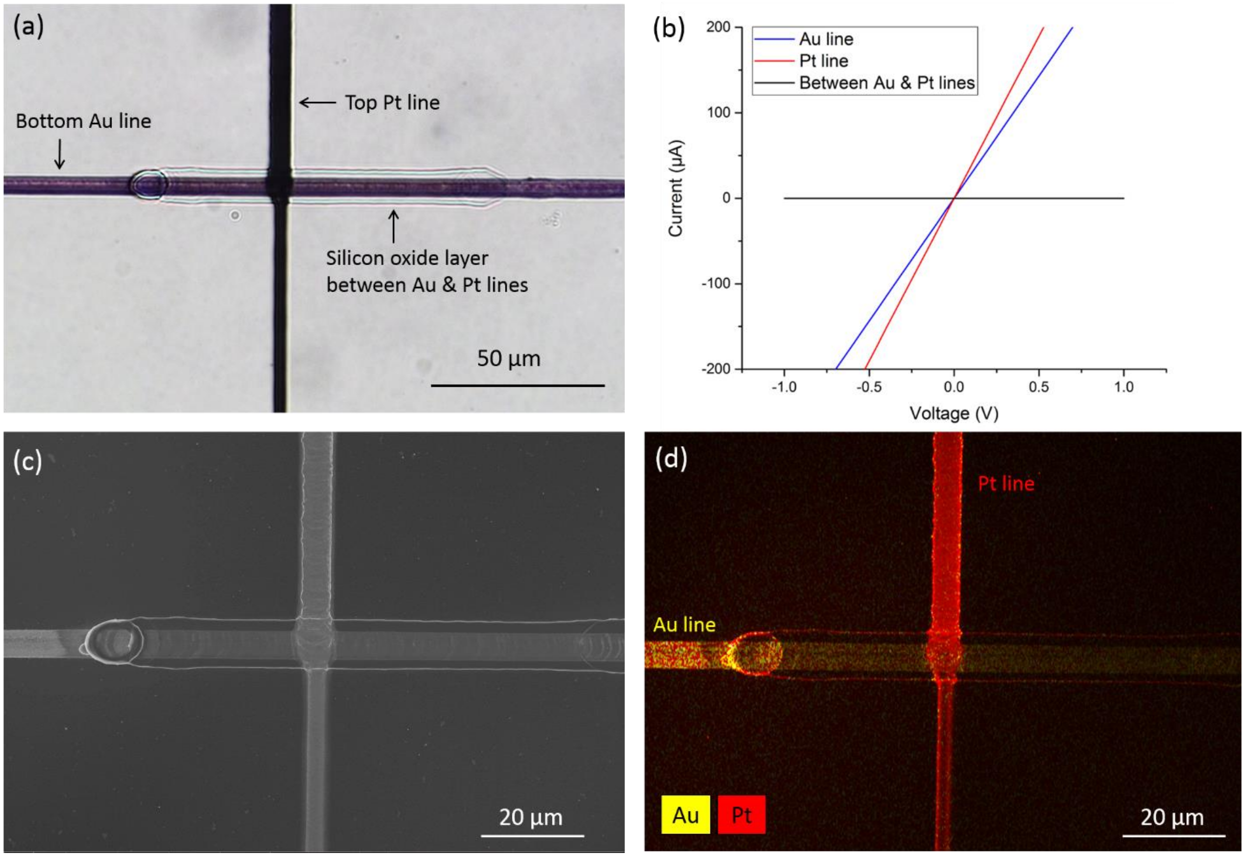

Figure S5. (a) Bright-field microscopy image and (b) corresponding I-V measurements of an Au/silicon-oxide/Pt junction. (c) HR-SEM image and (d) EDS mapping of Au and Pt.

Video SV1: shows the fabrication of a conducting/insulating/conducting multi-layered micro-structure using 3 wt% of Pt based precursor for the two conducting layers and TEOS based precursor for the insulating layer. The laser power was 14 mW, and the stage velocity was 100 and 400 µm/s for Pt and silicon oxide deposition, respectively.

Video SV2: demonstrates micro-printing in a microfluidic channel. Switching of precursors (Pt and TEOS based) was obtained by a microfluidic pressure-based controller. At the end of this video, a HR-SEM image of the fabricated cross-sectioned micro-structure is displayed.
